# P310: The blood accidents exposure (aes) in principal hospital dakar (hpd): about 152 cases collected in 12 years

**DOI:** 10.1186/2047-2994-2-S1-P310

**Published:** 2013-06-20

**Authors:** PS Ba, B Fall, FK Soumah-Mbaye, KM BA-Fall, PS Mbaye

**Affiliations:** 1 Medicine Department , HPD, Dakar, Senegal; 2 Federation laboratories, HPD, Dakar, Senegal

## Introduction

Blood exposure accidents (BEA) are a risk to all personnel of the health services. Today, support for BEA is well codified. In Senegal, a strategy implemented by ISAARV provides rapid and optimized response to these accidents, in order to reduce the risk of HIV seroconversion.

## Objectives

- Specify frequency, circumstances and place of occurrence of BEA among staff, - Highlight the attitudes and practices of personnel during blood exposure situations, - Evaluate the management of these accidents.

## Methods

Retrospective study of all cases reported to the BEA Principal Hospital in Dakar during the period January 2001 - December 2012. Reporting cases of BEA was made in a register in health service staff with collection of a declaration. Declaration forms of all cases were compiled and analyzed in Epi Info version 6.4.

## Results

One hundred and fifty two (152) cases of BSE have been reported and were taken care of. All personal health services were concerned with a predominance of nurses (44.1%). More than half (59.6%) were vaccinated against hepatitis B at the time of the accident. Needlestick injuries were the most frequently encountered accidents (86.5%) followed bz the taking of samples (44.1%) or the placement of infusions (22%). Only 55.9% of the victims were wearing gloves at the time of the accident. After the accident, 49.2% of victims washed their hands and used disinfectants recommended in case of BEA. The source patient was identified in 78% of cases, with15 cases of HIV positive patients. In the evaluation, 88.1% were intermediate risk of BEA. Chemoprophylaxis was introduced in 84.7% of cases, treatment did not exceed 2 days except for 13 of the 15 victims of BEA whose source patients were HIV positive at the time of the accident. No seroconversions were noted.

## Conclusion

This study has made it possible to make recommendations for effective prevention of the BEA, in a broader context of risk management and safety at work.

## Disclosure of interest

None declared

